# Ethical Pitfalls in AI‐Based Predictive Models in Surgery

**DOI:** 10.1002/wjs.70080

**Published:** 2025-09-04

**Authors:** Sara Ben Hmido, Houssam Abder Rahim, Boris Keller, Freek Daams, Matthijs Schakel, J. Carel Goslings, E. J. M. Nieveen van Dijkum, Stephen Rainey, Geert Kazemier, Marieke Bak, Corrette Ploem

**Affiliations:** ^1^ Department of Surgery Amsterdam University Medical Centers Amsterdam the Netherlands; ^2^ Cancer Center Amsterdam Amsterdam the Netherlands; ^3^ TU Delft Delft the Netherlands; ^4^ Department of Epidemiology and Data Science Amsterdam University Medical Centers Amsterdam the Netherlands; ^5^ Trauma Unit OLVG Hospital Amsterdam the Netherlands; ^6^ Philosophy Department of Values Technology and Innovation (VTI) Delft University of Technology Delft the Netherlands; ^7^ Department of Ethics Law and Humanities Amsterdam UMC (Location AMC) University of Amsterdam Amsterdam the Netherlands

**Keywords:** AI, complications, ethics, machine learning, prediction, stakeholders, surgery

## Abstract

**Background:**

Predictive models in surgery promise to improve clinical care by anticipating complications, guiding decision‐making, and supporting personalized treatment strategies. Although their potential to enhance outcomes and efficiency is substantial, their integration into clinical practice also raises profound ethical challenges.

**Ethical Framework:**

These challenges span the entire lifecycle of predictive models from data collection and development to validation and clinical use. They touch upon patient privacy, algorithmic bias, transparency, and the shifting responsibilities of clinicians. Importantly, the ethical concerns are not isolated to one group but shared across patients, developers, and clinicians within a dynamic stakeholder relationship.

**Analysis:**

Key risks include biased or unrepresentative datasets, privacy breaches, opaque decision‐making processes, and the danger of deskilling surgeons if reliance on algorithms becomes excessive. To mitigate these risks, strategies, such as out‐of‐distribution detection, standardized data collection, parallel model development, and continuous auditing, are essential. Beyond technical safeguards, embedding predictive models within a framework of accountability and patient‐centered care is necessary to sustain trust and equity.

**Conclusion:**

The integration of predictive models into surgery requires more than technical excellence, and it demands ethical vigilance. Preparing future clinicians through education that emphasizes both clinical reasoning and ethical awareness is critical. By aligning predictive model development with human‐centered values, healthcare systems can ensure that these innovations enhance surgical practice while safeguarding equity, transparency, and patient trust.

## Graphical abstract

Predictive models in surgery promise to improve clinical care by anticipating complications, guiding decision‐making, and supporting personalized treatment strategies. Although their potential to enhance outcomes and efficiency is substantial, their integration into clinical practice also raises profound ethical challenges. These challenges span the entire lifecycle of predictive models from data collection and development to validation and clinical use. They touch upon patient privacy, algorithmic bias, transparency, and the shifting responsibilities of clinicians.

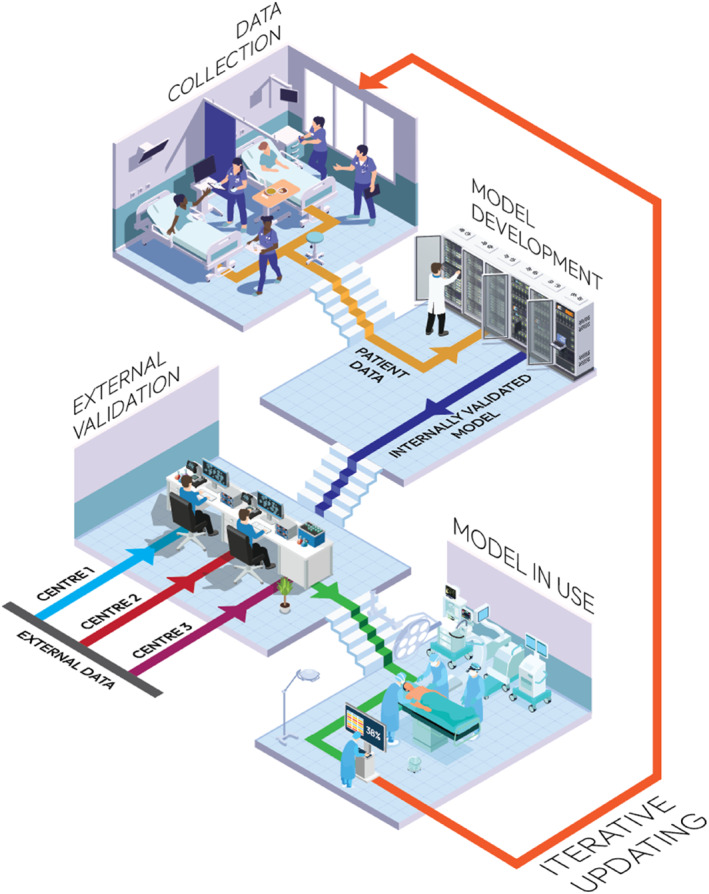

## 1 Introduction

Anastomotic leakage (AL) is one of the most severe postoperative complications in gastrointestinal surgical care, with significant impacts on patient outcomes, morbidity, and healthcare costs [1, 2]. To address this complication, predictive models have been developed to assess the risk of AL, representing a critical step toward improving surgical planning and postoperative management [3]. By leveraging patient data and advanced algorithms, models can serve as clinical decision support tools, offering personalized insights to enable more informed and proactive decision‐making.

Beyond AL, predictive models have also been designed as clinical decision support systems to address complications, such as surgical site infections and quality of life outcomes, including low anterior resection syndrome [3, 4, 5]. These tools demonstrate the transformative potential of artificial intelligence in enhancing surgical care, reducing preventable errors, and improving overall care quality. However, alongside these advancements, the adoption of predictive models introduces significant ethical challenges that demand careful consideration at every stage of their lifecycle [6].

Development and use of predictive models in surgery involve multiple stages, each presenting unique ethical challenges, also reflected in the ‘Ethical Principles for Trustworthy AI’ of the EU [7]. During data collection, issues in data collection and processing, protection of medical confidentiality, and bias must be addressed, the latter to ensure equitable and robust model performance. Especially in the model's developmental phase, decisions about prediction methods and validation determine its accuracy and generalizability. In the phase of introducing AI‐based predictive models in practice, issues may arise concerning the decision‐making process, such as reduced patient‐centered care, ambiguity about the clinicians' responsibilities and accountability, potential deskilling of surgeons, and the like.

The cyclical process of patients generating outcome data, developers improving predictive capabilities, and clinicians applying these tools calls for rigorous collaboration and critical ethical evaluation. This paper identifies the main ethical issues of relevance to developers, users, and patients involved in the introduction of AI‐based predictive models in surgery and discusses strategies for dealing with them. These issues need to be addressed in time to prevent that AI systems will be marketed or used by surgeons that are unethical. However, before we do so, we briefly picture the process of AI‐based model development in general and indicate which parties are involved in the different phases and what their most important roles are.

## 2 Predictive Modeling in Surgery: A Cyclical Process

As pointed out in the growing literature, AI‐based predictions promise to be helpful in many areas of medicine [8]. A good example is the field of surgical interventions AI‐models generating information which is useful for anticipating the risks and outcomes of surgical procedures. Their output assists with diagnosing and screening the patient and supports both clinician and patient in deciding about surgical interventions and the necessary preoperative measures [9]. Predictive models enable personalized data‐driven decisions that enhance treatment outcomes [10].

**FIGURE 1:**
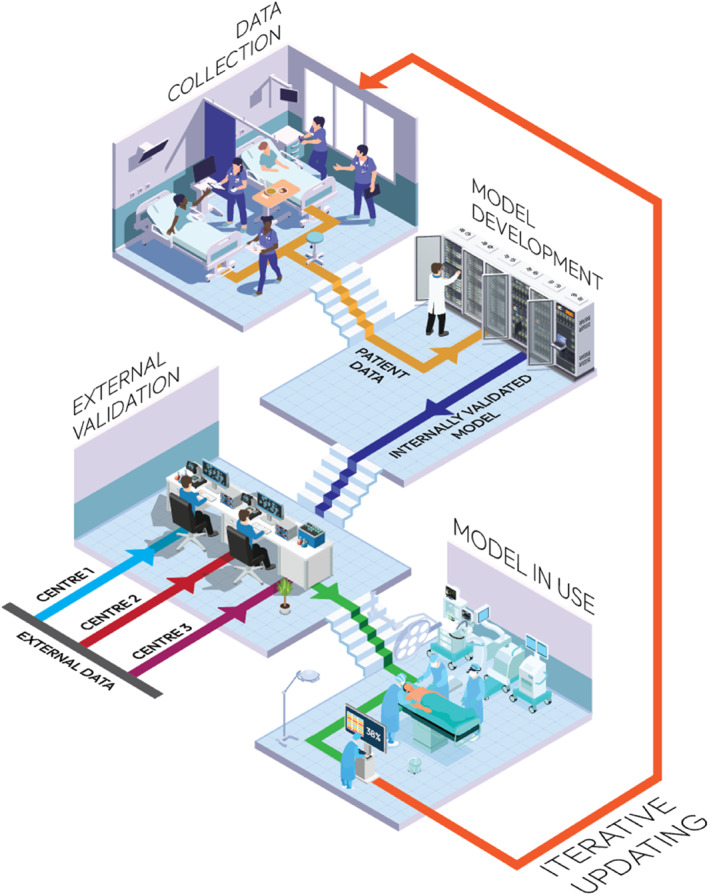
Infographic of the lifecycle of a predictive model.

A good and reliable AI model within surgery that assumes these advantages is not easily achieved and requires a solid development process. It should cover all the key steps for good AI‐model development, starting with data collection and model development and ending with validating and updating the model. We regard the developmental process of such AI‐based models as a cyclical process [11]. The infographic below (Figure 1) outlines the models' lifecycle.

**FIGURE 2:**
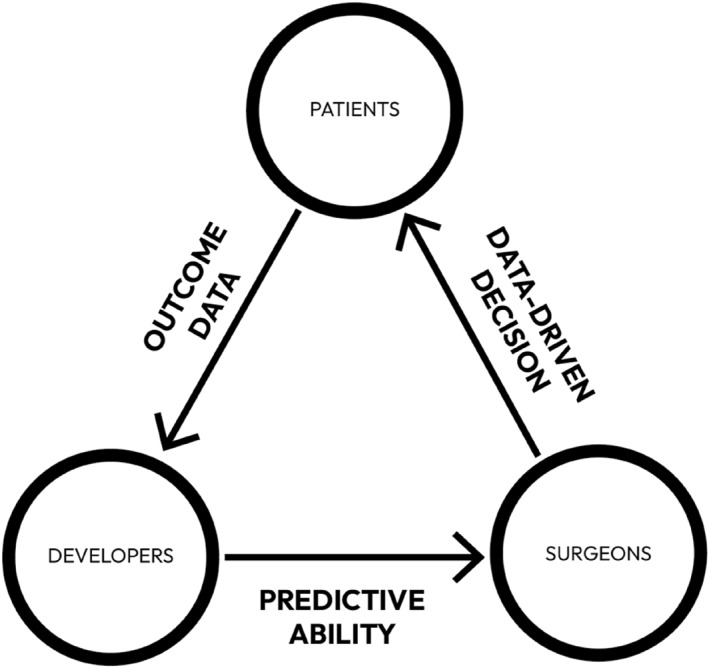
The cyclical relationship between patients, developers, and healthcare professionals.

Within the lifecycle of any AI‐based model, three key stakeholders are involved as follows: patients, developers, and clinicians. We consider their relationship as a ‘stakeholder triangle’ (Figure 2). Essentially, within this triangular relationship, patients provide their data, developers create algorithms on the basis of these data, and clinicians, that are in our case mainly surgeons, apply the algorithms and use their output to make clinical decision‐making better informed [12].

Each of the stakeholders has its own interests and concerns in relation to the development and use of these predictive models. For *patients*, the primary concerns are their data and privacy being well protected and receiving care that is safe and of high quality [13]. However, reliance on algorithms introduces uncertainties distinct from traditional clinical judgment, raising concerns about accuracy and trust [14].

*Developers* focus on creating reliable and fair models that predict surgical outcomes effectively and pose the least risk of product liability after they are marketed. They must ensure equitable performance across all patient groups and address biases. Postdeployment, developers maintain model reliability by monitoring data shifts, mitigating model drift, and updating algorithms as needed. Developers should ensure that predictive models are guided by clear clinical needs. A lifecycle perspective includes not only postdeployment monitoring but also early consideration of whether development is warranted.

*Clinicians* rely on predictive insights to refine their decisions, improving patient outcomes. However, they must interpret model outputs within each patient's unique context and take into account their patients' individual preferences. Balancing data‐driven predictions with clinical expertise ensures that decisions remain patient‐centered, prioritizing safety and efficacy.

The following paragraphs explore key ethical issues in developing and using AI‐based surgical prediction models. Aligning these tools with ethical standards is essential as failure to address such concerns could lead to flawed models or substandard care.

## 3 Key Ethical Issues in the Data Collection

Although patients are presumably intended as the ultimate beneficiaries of predictive models, they also serve as the starting point for any model development. Patient data are considered as the foundational basis for these models acting as the “seed” from which predictions are derived.

This raises ethical concerns, particularly around privacy, informed consent, and the potential for data misuse. Ensuring that patient data are used responsibly is critical as any bias or inaccuracy in the data can negatively impact the fairness and reliability of the model's outcomes.

### 3.1 Potential for Data Misuse

Despite safeguards, the potential for data misuse remains a serious concern [13, 15]. Anonymized datasets can be reidentified, especially when combined with other datasets [16]. This risk becomes especially pressing as predictive models and their data increasingly shift from hospitals and research institutions to commercial entities, where motivations are also profit‐driven.

Misuse of predictive models can arise not just from data issues but from how they are applied. For instance, AL prediction models meant to aid surgery could be repurposed by insurers to assess financial risk, leading to higher premiums or denied coverage based on algorithmic probabilities rather than clinical need.

A statement from the American Medical Association (AMA) warned that health insurers increasingly use AI to automate prior authorization denials, often without clinician oversight, and sometimes overriding medical judgment [17]. In practice, this has resulted in patients being denied medically necessary surgeries or postoperative care based on predictive outputs, raising profound ethical and legal questions [18]. Such actions may disproportionately impact individuals with preexisting conditions or marginalized demographics, compounding existing healthcare inequities. Moreover, because these models infer risk based on population data rather than individual nuance, their outputs are probabilistic not deterministic—yet insurers may treat them as definitive unjustly penalizing patients.

### 3.2 Bias Arises or an Existing Bias Is Magnified

Bias in predictive models can originate during the data collection stage, where choices about what data to gather, from whom, and in what setting can significantly impact model fairness and reliability.

In the context of AL, for example, models trained primarily on data from one hospital group may not generalize well to another hospital group in another jurisdiction, where differences in surgical techniques, patient demographics, or resource availability can influence outcomes. Beyond these clinical factors, discrepancies in how hospitals record data or define complications, such as AL, can further affect model performance. One institution may use strict diagnostic criteria and structured electronic health records (EHRs), whereas another relies on subjective assessments, leading to inconsistencies in labeling and risk estimation. If a model is trained on data with such discrepancies, it may misclassify patients when applied elsewhere, reinforcing biases rather than mitigating them. These risks are particularly pronounced in low‐resource or global settings, where infrastructure and data collection standards may differ significantly from those of the original development context.

A well‐documented example of bias in surgical predictive modeling comes from a 2024 study on postoperative complication prediction for total joint arthroplasty. The model performed well for non‐Hispanic White patients but significantly underperformed for minority groups due to their underrepresentation in the training data [19]. This resulted in poor generalization and risk misestimation for these populations. Such disparities can lead to inequitable clinical decisions, such as failing to identify high‐risk cases or overestimating risk inappropriately, thereby amplifying existing inequalities in surgical care.

### 3.3 Potential Strategies to Address Ethical Challenges in Data Collection Out‐of‐Distribution Detection

To mitigate bias in predictive models, efforts should start with diverse and representative data collection, inclusive sampling methods, and regular audits. Collecting data that reflects a wide range of demographics and clinical profiles is essential for equitable model performance across populations. However, true representativeness can be challenging and sometimes unattainable due to systemic biases, data limitations, and hard‐to‐capture populations, making some level of bias inevitable.

To address this, out‐of‐distribution (OOD) detection could be implemented alongside predictive models [20]. This mechanism helps clinicians assess how closely a patient's characteristics align with the model's training and validation data. Technically, this can be achieved by analyzing whether a patient's profile falls within the standard deviation of the training dataset. If a profile is flagged as out‐of‐distribution, it serves as a warning that the model's predictions may be less reliable and should be interpreted with caution. Researchers are actively developing methods to improve OOD detection and its application in clinical practice [21].

### 3.4 Data Standardization

Standardizing healthcare data are essential for building reliable and unbiased predictive models. Inconsistencies in EHRs, such as varying naming conventions, formats, and definitions, can introduce bias and reduce model accuracy. These discrepancies become more pronounced across institutions, hindering data integration and generalizability.

Frameworks, such as Health Level 7, SNOMED CT, and LOINC, provide tools to address these issues by standardizing healthcare data [22, 23, 24]. HL7 facilitates data exchange, SNOMED CT provides uniform clinical terminology, and LOINC standardizes observations. To address these challenges, healthcare organizations must prioritize the consistent and comprehensive adoption of these standards. Doing so will create the foundation for predictive models that are reliable, scalable, and equitable.

## 4 Key Ethical Issues in the Development of the Model

The development phase of predictive models introduces acute ethical challenges, particularly in ensuring fairness, transparency, and accountability.

Decisions made during model design, such as choosing algorithms, selecting features, and setting parameters, can unintentionally lead to unequal or discriminatory outcomes once the application has been put into practice.

Furthermore, the complexity of many models can limit their interpretability, making it difficult for practitioners to understand or explain the basis for certain predictions. Ensuring that the development process is technically and ethically sound requires ongoing scrutiny to prevent unintended harm, prioritize patient welfare, and align the model's design with core healthcare ethics.

### 4.1 Selecting the Prediction Method

Predictive models range from simpler statistical techniques, such as logistic regression, to advanced artificial intelligence methods such as artificial neural networks (ANNs) and random forests (RFs). Each has distinct advantages depending on data complexity and analysis goals, making the selection process challenging. Factors, such as dataset size, interpretability, computational resources, and team expertise, all influence this choice. Importantly, interpretability remains crucial in healthcare, where transparent models, such as logistic regression, can be beneficial.

In a study by Taha‐Mehlitz et al. (2024), six modeling techniques, including logistic regression and artificial intelligence methods, were tested for performance [5]. Surprisingly, logistic regression slightly outperformed random forests in area under the curve (AUC) discrimination, demonstrating that simpler models can sometimes match or exceed complex ones.

More complex models, such as ANN and RF, although capable of handling intricate data, often function as “black boxes,” where the processes underlying their predictions are opaque. This lack of transparency poses challenges in healthcare as it hinders the ability of clinicians and patients to fully understand and trust the decision‐making process. Such opacity can compromise patient autonomy and make informed decision‐making more difficult [25].

### 4.2 Appropriately Handling Internal and External Validation

Rigorous internal and external validation is essential before implementing a predictive model to ensure its reliability, accuracy, and generalizability. Internal validation assesses the model within its original dataset, often using cross‐validation, to detect overfitting and confirm consistency. However, internal validation alone is insufficient as models must also perform reliably in new environments.

External validation tests a model across diverse populations and settings to ensure it generalizes beyond its development group and helps uncover bias (see Section 3.2). Without it, models may fail in different contexts—for example, one trained on urban patients may misjudge risk in rural areas leading to harmful delays or exclusions. These challenges are even more critical in low‐resource settings, where local healthcare realities may differ substantially and the risk of out‐of‐distribution application is higher. Broad validation is key to accuracy, equity, and reliable clinical use.

It is also important to recognize that validation only provides a “snapshot” of performance at a given time as highlighted by Van Calster et al. (2023) [26]. Over time, changes in clinical practices or patient populations, patient demographics, and technology may alter the model's performance, necessitating ongoing monitoring, revalidation, or updating to maintain relevance in dynamic healthcare settings.

All considerations from the data collection phase must also be revisited and often with greater scrutiny. Since external datasets are not created internally, there is limited visibility into how the data were collected, by whom, and under what conditions.

### 4.3 Potential Strategies to Address Ethical Challenges in Development

#### 4.3.1 Parallel Model Development

A potential solution to selecting the optimal predictive model is a parallel approach, where developers create and test multiple models, such as LR, RF, and ANN, on the same dataset as demonstrated by Taha‐Mehlitz et al. (2024).5 This method allows developers to evaluate each model's interpretability, accuracy, and robustness, helping to identify which model fits the specific healthcare application best. By developing models side‐by‐side, developers can gain a comprehensive view of each model's strengths and limitations, enabling more informed choices that align with both the technical requirements and clinical objectives of the project. This process is most effective when grounded in a clearly defined clinical question, ensuring that model development responds to genuine needs rather than being driven by technical interest alone.

#### 4.3.2 Continuous Auditing and Self‐Validation Tools

To ensure predictive models in healthcare remain reliable, accurate, and ethical, continuous auditing is essential beyond initial validation stages. As clinical practices, populations, and technologies evolve, both healthcare institutions and model developers have roles to play in ongoing monitoring. Although large hospitals may be equipped to perform internal audits, such as quarterly performance reviews or daily drift detection, smaller clinics and low‐resource settings may lack the capacity to implement these processes.

To support consistent and safe use across varied environments, developers should provide accessible tools for local validation, recalibration, and performance monitoring. These tools could include features such as automated alerts for performance changes, user‐friendly interfaces for reevaluation, and clear guidance for maintaining model relevance over time. Providing such support aligns with regulatory frameworks, such as the EU AI Act and MDR, and helps uphold ethical principles by identifying biases, preventing harm, and ensuring models remain appropriate for the populations they serve.

By enabling healthcare providers in all settings to maintain oversight of predictive models, developers contribute to a system of shared responsibility that promotes trust, safety, and accountability in AI‐supported care.

## 5 Key Ethical Issues in the Decision‐Making

In the predictive model cycle, the most critical potential failure occurs in the leg between patients and surgeons, specifically in the application of data‐driven decisions. This is the point where predictive insights generated by the model are used by the medical team to make decisions about the patient's care. However, as Sauerbrei et al. argue, they must do so while simultaneously ensuring shared decision‐making and patient‐centered care [27].

### 5.1 Balancing Algorithmic Guidance With Clinical Judgment

Predictive models offer promise for personalized care through data‐driven recommendations but overreliance on them may overlook individual patient contexts. Although these tools analyze broad trends, they cannot fully account for unique factors such as medical history or social determinants. Surgeons must integrate model insights with their expertise to maintain patient‐centered care.

For example, a model predicting AL risk may recommend avoiding a surgical approach based on general trends but fail to consider patient‐specific factors such as prior surgeries or intraoperative findings. Blind adherence to such recommendations could lead to unnecessary changes in surgical plans.

Overreliance on models can shift accountability from clinicians to opaque algorithms creating responsibility gaps. Maris et al. (2024) highlight patients' concerns about losing the “human touch” in AI‐driven healthcare settings, emphasizing the irreplaceable role of clinicians in interpreting and assessing algorithmic recommendations [28]. They also advocate for normative research on patients’ “right to a human doctor,” reinforcing the importance of empathy, individualized care, and accountability in decision‐making.

Accountability must stay with the surgeon, who is best equipped to balance algorithmic advice with clinical and ethical judgment. Overreliance on predictive models risks eroding patient trust and violating the duty of care, especially if model errors cause harm. Transparency about system limitations and shared decision‐making are essential to maintain trust and uphold ethical standards.

By reinforcing accountability, fostering shared decision‐making, and safeguarding the “human touch,” clinicians can ensure that predictive models enhance patient care rather than compromise its core humanistic and ethical principles.

### 5.2 Deskilling the Future Generation of Surgeons

There is another crucial phenomenon that these models bring about within clinical settings: the creeping and pervasive risk of deskilling among surgeons. Overreliance on model outputs can lead to a diminished capacity for critical diagnostics. The danger lies in being cognizant of the models ‘superiority and not being acquainted with the digital decision making process. Rainey et al.’s (2022) warn of the dangerous perception of AI as a “superior” decision‐maker, a belief that could lead practitioners to uncritically accept algorithmic guidance [29].

For example, if surgeons rely too heavily on an AI model predicting AL risk, they may overlook critical intraoperative factors such as tension or vascularization. Over time, this could erode their independent judgment, a deskilling effect that becomes risky if the model degrades or underperforms in certain patient groups.

A current real‐world example of deskilling concerns is seen in diagnostic radiology, where a qualitative study by Chen et al. (2021) has shown that AI integration may lead to an unconscious reliance on algorithmic tools [30]. As AI improves in detecting imaging abnormalities, radiologists risk losing the ability to identify nuanced features outside AI parameters. This reliance becomes problematic if AI performance falters, reducing their diagnostic adaptability. Chen et al. stress the need for radiologists to maintain expertise alongside AI to ensure resilience and avoid potential liability risks.

### 5.3 Potential Strategies to Address Ethical Challenges in Decision‐Making

#### 5.3.1 Educating Future Generations

Both the loss of patient‐centered care and the risk of deskilling arise from overreliance on predictive models. To address this, clinicians should be encouraged to develop and document their clinical impressions alongside model predictions rather than relying solely on algorithmic output. Although in the future it may not be practical to separate human and machine input entirely, especially as predictive tools become embedded in workflows and improve efficiency, the emphasis should remain on maintaining clinical reasoning as a core skill. Positioning models as advisors rather than decision‐makers helps ensure that clinicians stay actively engaged in interpretation, judgment, and care delivery.

As predictive models and AI become standard in healthcare, future medical professionals must learn to use these tools responsibly. For instance, surgeons using an AI‐driven AL prediction model may receive basic training on its usage but often lack in‐depth knowledge of how it works, what biases exist in its training data, and where its limitations lie. This raises the question: Do they need to fully understand the model's internal workings or is it enough to know how to interpret and apply its outputs? Not every surgeon needs to be a data scientist, just as a driver does not need to be an engineer to operate a car. Still, clinicians must understand the limitations of predictive models and recognize how and when they might fail. Equally important is knowing how to interpret model outputs and integrate them into their own clinical judgment, especially when deciding whether a model is appropriate for a given context. This reinforces the irreplaceable role of human expertise in clinical decision‐making.

Currently, many medical students have limited skills and exposure when it comes to engaging with AI tools, which can leave them underprepared [31]. Educational programs should teach students how to use predictive models and other AI tools ethically and responsibly, with a clear understanding of their capabilities and limitations. By incorporating AI into medical education, future clinicians can develop a balanced approach to technology that supports rather than undermines core medical knowledge. AI should reinforce foundational principles and clinical reasoning, not replace them, ensuring that students remain actively engaged in their learning. This approach encourages critical thinking, patient‐centered care, and continuous skill development in an AI‐enhanced environment.

## 6 Concluding Remarks

The integration of AI‐based predictive models into surgical practice holds tremendous promise for enhancing patient care, reducing complications, and improving outcomes. However, as with any transformative technology, the development and use of these tools are accompanied by significant ethical challenges. Issues, such as bias, data misuse, diminished transparency, and the potential for deskilling clinicians, highlight the need for careful consideration and proactive mitigation strategies. An overview of these pitfalls, their underlying causes, and possible solutions is presented in Table 1.

**TABLE 1:** Overview of the ethical pitfalls, their underlying cause, and potential solutions.

Pitfall	Cause	Potential solution
Potential for data misuse	Anonymized datasets can be reidentified when combined with other data, particularly by commercial entities.	Enforce strict legal and regulatory safeguards, mandate the use of robust anonymization techniques, and implement transparency policies requiring disclosure of data‐sharing arrangements.
Bias in data collection	Sampling from nonrepresentative populations or using biased proxies, such as healthcare costs, can result in unfair predictions that disproportionately impact specific groups.	Use diverse and representative data sampling. Implement out‐of‐distribution (OOD) detection mechanisms to flag cases where a patient's characteristics differ from the model's training data. Conduct regular audits of dataset fairness and representativeness.
Lack of data standardization	Variability in healthcare documentation practices and terminologies across institutions leads to inconsistent data integration.	Adopt and enforce standardized frameworks, such as HL7, SNOMED CT, and LOINC, for data collection and exchange. Provide training for healthcare staff to ensure consistent data entry practices.
Choosing the prediction method	Overreliance on complex “black‐box” models, such as neural networks, can reduce interpretability and transparency, compromising trust and accountability.	Use a parallel model development approach to evaluate multiple methods (e.g., logistic regression vs. neural networks) for accuracy, transparency, and suitability. Prioritize interpretable models where possible, especially in high‐stakes applications such as surgery.
Validation challenges	Internal validation alone may lead to overfitting, whereas external validation can be hindered by differences in data quality or collection methods across institutions.	Conduct both internal and external validation on diverse datasets. Use continuous auditing to monitor performance over time. Provide hospitals with self‐validation tools to assess the relevance of models in real‐world applications.
Algorithmic overreliance	Clinicians may rely excessively on predictive models, reducing accountability, and neglecting individual patient contexts.	Encourage independent clinical assessments alongside model recommendations. Require clinicians to document their reasoning when using predictive insights.
Deskilling of clinicians	Prolonged use of predictive models can lead to diminished diagnostic and decision‐making skills among medical practitioners.	Integrate AI and predictive model training into medical education. Encourage active engagement with predictive tools while emphasizing the importance of independent diagnostic reasoning. Develop exercises to test clinicians' skills without model input periodically.

These challenges, although serious, are not insurmountable. Through effective stakeholder collaboration that bridges the perspectives of patients, developers, and surgeons, practical application can be established. Prioritizing transparent data practices, rigorous validation, and accountability ensures equity, trust, and patient‐centeredness, aligning predictive models with core bioethical principles.

Moreover, as predictive models become routine in surgical decision‐making, the importance of preparing future generations of surgeons cannot be overstated. Medical education must evolve to teach clinicians not only how to interpret and apply predictive models responsibly but also how to recognize their limitations. Balancing the use of these tools with clinical expertise will help sustain critical diagnostic skills, preserve the human touch in patient care, and uphold shared decision‐making principles.

By embedding ethical vigilance and a culture of continuous learning into the lifecycle of predictive models, the surgical community can maximize their benefits while minimizing potential harms. This commitment will ensure that AI‐driven innovations enhance surgical practice without compromising the values and principles that define high‐quality patient care.

## Author Contributions

Conceptualization: Sara Ben Hmido, Houssam Abder Rahim, Boris Keller. Funding acquisition: Freek Daams. Investigation: Sara Ben Hmido, Houssam Abder Rahim, Boris Keller. Methodology: Sara Ben Hmido, Houssam Abder Rahim, Boris Keller, Corrette Ploem. Supervision: Corrette Ploem, Freek Daams, Marieke Bak. Visualization: Houssam Abder Rahim. Writing – original draft: Sara Ben Hmido, Houssam Abder Rahim, Boris Keller. Writing – review and editing: Freek Daams, Matthijs Schakel, J. Carel Goslings, E. J. M Nieveen van Dijkum, Stephen Rainey, Geert Kazemier, Marieke Bak, Corrette Ploem.

## Conflicts of Interest

All other authors declare no conflicts of interest. Medtronic had no role in study design, data collection, data analysis, data interpretation, or writing of the manuscript.

## Data Availability

Data sharing is not applicable to this article as no datasets were generated or analyzed during the current study.
